# Gender-Diverse Youth with Turner Syndrome: Special Management Considerations

**DOI:** 10.1210/jcemcr/luae076

**Published:** 2024-05-03

**Authors:** Kelsey B Eitel, Anna Zenno, Carolina Di Blasi, Patricia Y Fechner, Juanita K Hodax

**Affiliations:** Department of Pediatrics, University of Washington, Seattle, WA 98105, USA; Department of Pediatrics, University of Washington, Seattle, WA 98105, USA; Department of Internal Medicine, University of Washington, Seattle, WA 98105, USA; Department of Pediatrics, University of Washington, Seattle, WA 98105, USA; Department of Pediatrics, University of Washington, Seattle, WA 98105, USA; Department of Pediatrics, University of Washington, Seattle, WA 98105, USA

**Keywords:** Turner syndrome, gender-diverse youth, adolescents, testosterone, gender-affirming hormones, hypogonadism

## Abstract

Turner syndrome (TS) is a sex chromosome abnormality characterized by short stature and primary hypogonadism with increased risk for cardiovascular disease, osteopenia, metabolic syndrome, diabetes mellitus, abnormal liver enzymes, and impairment of nonverbal learning skills. Gender-diverse youth include youth who have a gender identity that is different from their sex assigned at birth. They have an increased risk of suicidality, which is decreased in those who receive gender-affirming care. There have been no prior reports on the association or management of gender-diverse youth with TS. We describe 3 cases of gender-diverse youth with TS that highlight the importance of discussing gender identity in patients with hypogonadism in need of sex hormone replacement. Goals of care should be discussed to determine whether estrogen or testosterone replacement aligns best with gender identity. If a patient chooses to start testosterone, special considerations of risks such as erythrocytosis, osteopenia, and cardiovascular disease should be discussed in relation to their TS.

## Introduction

Turner syndrome (TS) is a sex chromosome abnormality that affects 25 to 50 per 100 000 of those assigned female at birth (AFAB) (1). It is characterized by short stature and hypergonadotropic hypogonadism with increased risk for cardiovascular disease (CVD), osteopenia, metabolic syndrome, diabetes mellitus, abnormal liver enzymes, and impairment of nonverbal learning skills (1). Most patients with TS require sex steroid replacement for induction of puberty, maintaining secondary sex characteristics, normalizing uterine growth, attaining and maintaining peak bone mass, cardiovascular health, and cognitive function (2). TS management guidelines recommend starting low-dose estradiol at 11 to 12 years old if there is a lack of spontaneous puberty and biochemical confirmation of hypergonadotropic hypogonadism, with estradiol dose increases over 2 to 3 years (1). There is no discussion of testosterone use for gender-diverse patients (2).

In the United States, 1.8% of youth identify as transgender and have a higher prevalence of suicidality, substance use, and violence victimization (3). Gender-diverse youth receiving gender-affirming care have 60% lower odds of depression and 73% lower odds of suicidality (4). There have been no prior reports on the association or management of gender-diverse youth with TS. Special considerations for gender-diverse youth with TS include optimizing linear growth and bone mass accrual and minimizing CVD risk and mental health comorbidities. We describe 3 cases of gender-diverse youth with TS with varying goals of care and management strategies.

## Case Presentation

### Patient A

Patient A was AFAB with a history of TS, autoimmune hypothyroidism, anxiety, obsessive compulsive disorder, attention deficit hyperactivity disorder, and insomnia. TS was diagnosed prenatally with amniocentesis completed for advanced maternal age. Postnatal karyotype was 45, X[17]/46, X isodicentric (X) (p11.4)[13]. Growth hormone (GH) for short stature was delayed until 13 years and 10 months old due to needle phobia. Spontaneous thelarche occurred at 12 years old, although FSH was 71.7 IU/L (0.3-7.8 IU/L). He continually shared that he felt like a boy since he was in preschool.

### Patient B

Patient B was AFAB with a history of TS, autoimmune hypothyroidism, myopia, obesity, depression, and anxiety. TS was diagnosed at 8 years old during short-stature evaluation with karyotype of 46, X, psu idic(X)(p11.2). At 12 years old, an exam showed Tanner 2 breasts, and FSH was 32.5 IU/L. Female gender identity was presumed, and oral estradiol was started as the patient declined transdermal estrogen. At 13 years old, menarche occurred, and progesterone was added with subsequent transition to an oral contraceptive pill (OCP). The patient self-discontinued his OCP at 14 years old after identifying as male. At 15 years old, he disclosed a male gender identity to his endocrinologist after a discussion of restarting estradiol. He reported exploring his gender since he was 11 years old and, starting at 14 years old, had socially transitioned at home and school.

### Patient C

Patient C was AFAB with a history of TS, atrial septal defect closure, and fatty liver disease. TS was diagnosed at 16 years old during evaluation for primary amenorrhea with spontaneous thelarche. Karyotype was 46X, i(X)(q10). Transdermal estrogen was started followed by progesterone 2 years later. At 20 years old, menarche occurred, and they reported identifying as nonbinary.

## Diagnostic Assessment

### Patient A

He had a persistent goal of starting puberty with testosterone and was referred to the gender clinic at 13 years 10 months, although he did not schedule an appointment due to a family focus on optimizing height outcomes with GH and not feeling ready to start a sex steroid. He had socially transitioned with clothes and hairstyle but was waiting to change pronouns until he started testosterone. Regarding his mental health, he was taking a stimulant for attention deficit hyperactivity disorder, clonidine for insomnia, and a selective serotonin reuptake inhibitor (SSRI). His obsessive-compulsive disorder improved after exposure therapy and returning to in-person school after the COVID-19 pandemic. When he established with the gender clinic at 15 years and 10 months, he had persistent male identity and remained interested in starting testosterone with the goal of voice deepening, body shape changes, and increased muscle mass. His height was in the 7th percentile on the Centers for Disease Control and Prevention (CDC) girls and 0.66th percentile on the CDC boys charts. Bone age was 13 years by female standards, with chronological age of 15 years and 3 months. An exam showed Tanner 3 breasts, and FSH was 80.3 IU/L.

### Patient B

He was referred to the gender clinic and reported his goals were a masculine body shape, flat chest, and voice deepening. Regarding his mental health, he was on an SSRI and seeing a therapist and reported a prior history of self-harming and suicidal ideation. At 15 years and 8 months old, his bone age was 16 years old using the female standard.

### Patient C

They reported they were comfortable continuing on estrogen and wanted further time to explore their embodiment goals with a therapist.

## Treatment

### Patient A

After a discussion of risks and benefits of testosterone and informed consent by the patient and parent, he started subcutaneous testosterone 10 mg weekly. He established care with a therapist with gender expertise for further support around social and medical transition.

### Patient B

After a discussion of the risks and benefits of testosterone and informed consent by the patient and parents, he started subcutaneous testosterone 25 mg every 2 weeks as he had been off estradiol for over 1 year.

### Patient C

They continued a combined OCP with menses every 6 weeks.

## Outcome and Follow-up

### Patient A

Patient A is followed in the TS clinic and gender clinic with the goal of height optimization while on GH and testosterone. His most recent follow-up was 5 weeks after starting testosterone with height in the 9.4th percentile on the CDC girls and the 0.79th percentile on the CDC boys charts. Labs showed hematocrit 38.3% (0.38 L/L) (37.0-49.0%; 0.3-0.49 L/L), total testosterone by liquid chromatography-mass spectrometry 15 ng/dL (0.5 nmol/L) (3-303 ng/dL; 0.1–10.5 nmol/L for tanner 2), ALT 20 units/L (5–52 units/L). He continues to follow with his psychiatrist and therapist.

### Patient B

His subcutaneous testosterone dose was slowly increased. At follow up 21 months after starting subcutaneous testosterone, he was taking a dose of 30 mg weekly without breakthrough bleeding, improved depression symptoms with discontinuation of his SSRI, and no reported self-harm or suicidal ideation. Labs showed hematocrit 40.4% (0.4 L/L), trough total testosterone by liquid chromatography-mass spectrometry 41.8 ng/dL (1.45 nmol/L), total cholesterol 177 mg/dL (4.5 mmol/L) (125–200 mg/dL; 3.2–5.1 mmol/L), low-density lipoprotein 113 mg/dL (2.9 mmol/L) (63–130 mg/dL; 1.6–3.3 mmol/L), high-density lipoprotein (HDL) 50 mg/dL (1.29 mmol/L) (30–63 mg/dL; 0.78–1.6 mmol/L), alanine aminotransferase 24 units/L, hemoglobin A1C 5.1% (32 mmol/mol) (4.0–6.0%; 20–42 mmol/mol). He was working on healthy eating and exercising daily and lost 5 kilograms in the last month. His testosterone dose was further increased to 40 mg weekly to continue titration to an adult replacement dose. A dual x-ray absorptiometry scan will be obtained once he is on adult doses of testosterone.

### Patient C

Patient C transitioned care to an adult endocrinologist. They had a normal dual x-ray absorptiometry scan (total body Z-score −1.1 using the female standard). They have been followed for 2.5 years after disclosing their gender identity and remained comfortable staying on estrogen. They are continuing to explore their goals with a therapist and considering testosterone use to deepen their voice and have a flatter chest.

## Discussion

These are the first reported cases of gender-diverse youth with TS. These cases highlight the importance of discussing gender identity for all patients with hypogonadism in need of sex hormone replacement at the time of puberty induction and ongoing, especially with any concerns about medication adherence. For gender-diverse patients, goals of care should be discussed to determine whether estrogen or testosterone replacement aligns best.

Gender identity may be disclosed at any age, leading to different approaches to management. Gender-diverse youth of any age may benefit from social transition such as changing their name, pronouns, and clothing styles, in addition to seeing a therapist for mental health support. In early pubertal youth, age-appropriate discussions of the expected pubertal changes with estrogen and testosterone may help youth and their families make an informed decision on which sex steroid they will begin. Early and recurring discussions on gender identity and goals of care may help prevent delayed initiation of sex steroids and therefore delayed bone mass accrual. The age of sex steroid initiation is 11 to 12 years old in the TS guidelines (1). In guidelines for the treatment of gender-diverse youth, sex steroid initiation is recommended when the patient has the capacity to give informed consent, which is typically 13.5 to 16 years old (5, 6). Sex steroid initiation should be individualized and could reasonably occur at these ages in a patient with TS, persistent gender identity, and well-defined embodiment goals. Gender-diverse youth with TS are unlikely to benefit from a GnRHa as they are hypogonadal and at high risk for osteopenia. Gender-diverse youth with TS may have an improved growth rate from adding oxandrolone (1) and find the masculinizing effects favorable.

If a patient discloses their gender identity after being on adult doses of estradiol and is interested in the masculinizing effects of testosterone, then they can transition to an adult dose of testosterone using established guidelines (5, 6). Other gender-diverse patients may not desire the masculinizing changes from testosterone but may be interested in menstrual suppression to meet their goals. This can be achieved with estradiol and continuous progesterone, rather than cycling of progesterone (6).

For patients taking testosterone, special considerations of risks such as erythrocytosis, CVD, and osteopenia should be discussed in relation to their TS. Increasing hematocrit level is a common effect of testosterone and should be monitored every 3 months during the first year of therapy, then every 6 months thereafter (5, 6). Erythrocytosis is associated with tobacco use, long-acting undecanoate injections, and higher body mass index (BMI) (7). Erythrocytosis can cause hypertension and thromboembolic events (7), which further increases CVD risk in those with TS.

People with TS are at increased risk of aortic dissection and QTc interval prolongation, which requires monitoring of blood pressure, electrocardiogram, transthoracic echocardiography, and/or cardiac magnetic resonance scan based on established guidelines (1). Exogenous testosterone may increase cardiovascular risk through decreased HDL levels and a potential increase in BMI (9). Studies on testosterone use in gender-diverse adolescents have shown a small increase in BMI with no change in BMI SD score, decreases in HDL cholesterol, and no sustained changes in blood pressure (8, 9). However, studies in adults have shown decreased BMI after starting testosterone (10).

Exogenous testosterone does not increase hemoglobin A1c levels (9), and liver enzyme elevations are rare (5,6, 8‐10). The effects of testosterone on bone density in gender-diverse youth have shown significant increases in bone mineral density Z-scores after starting testosterone, although longer-term data on bone density in adults on testosterone is mixed (6, 11). There are no studies on the effects of testosterone in patients with TS.

We have described the first 3 cases in the literature of gender-diverse youth with TS. This emphasizes the need for eliciting gender identity and goals of care, special considerations for testosterone use, and the need for further research on the impacts of testosterone use in those with TS.

## Learning Points

- People with Turner syndrome may identify as gender diverse.
- Gender identity should be discussed at the time of pubertal induction and ongoing. Goals of care should be elicited to determine if estrogen or testosterone aligns best with gender identity.
- There are few risks for the use of exogenous testosterone in gender-diverse youth, although special considerations should be taken for those with Turner syndrome regarding bone mass accrual, final adult height, and CVD risk.

## Data Availability

Data sharing is not applicable to this article as no datasets were generated or analyzed during the current study.
